# Success Factors for Open Access

**DOI:** 10.2196/jmir.5.1.e1

**Published:** 2003-03-31

**Authors:** James E Till

**Affiliations:** ^1^University of TorontoDepartment of Medical Biophysics and Joint Centre for BioethicsToronto OntarioCanada; ^2^Ontario Cancer InstituteUniversity Health NetworkDivision of Epidemiology, Statistics and Behavioural ResearchToronto OntarioCanada

**Keywords:** Cybernetics, information dissemination, diffusion of innovation

## Abstract

Open access to the peer-reviewed primary research literature would greatly facilitate knowledge transfer between the creators and the users of the results of research and scholarship. Criteria are needed to assess the impact of recent initiatives, such as the Budapest Open Access Initiative. For example, how many open-access research journals exist within a given field, and what is the reputation of each one? And, how many openly-accessible institutional e-print archives have been created and how many are actually are being used by researchers and scholars? A simple approach to an assessment of the open-access portion of the medical literature is described, and some preliminary results are summarized. These preliminary results point to the need for incentives to foster the implementation of initiatives such as the Budapest Open Access Initiative. An example of an *incentive model* is proposed, where an agency or foundation that provides peer-reviewed grants-in-aid to researchers establishes an e-print archive. Only current grantees of the agency would be eligible to post reports about the results of research projects or programs that have been supported by the agency. Some advantages and implications of this particular model are outlined. It is suggested that incentive models of this kind are needed to increase the likelihood that open access to the primary medical research literature will soon reach a "tipping point" and move quickly toward wide acceptance.

## Introduction

One crucial way to provide online access to novel, valuable, and quality-assessed research reports is to foster open access to the peer-reviewed primary research literature. Examples of recent initiatives in this area are the Budapest Open Access Initiative (BOAI) [1] and the Free Online Scholarship movement [2].

To achieve open access to scholarly journal literature, the BOAI has recommended 2 complementary strategies [1]. The first is self-archiving, by researchers and scholars, of their refereed journal articles. The second is the fostering of open-access journals. Peter Suber, the editor of the Free Online Scholarship Newsletter [2], has argued that various objections (eg, that open access to scientific journal literature requires the sacrifice of peer review, revenue, copyright protection, or other strengths of traditional journals) are based on misunderstandings [3].

## What criteria could be used to evaluate the success of the BOAI?

Several criteria that could be used to evaluate the success of the BOAI have been summarized [4]. For example, one indicator, oriented toward the first of its strategies (self-archiving), is the number of universities (or analogous organizations, such as research institutes or networks) that have created institutional e-print archives and have adopted policies that encourage faculty to use them. A comprehensive list of various kinds of e-print archives has been assembled by the Virtual Technical Reports Center of the University of Maryland Libraries [5].

Another indicator, oriented toward the second BOAI strategy (more open-access journals), is the number of journals in each research field that are committed to open access, coupled with measures of the reputation or impact of each journal. A list of open-access medical journals is available from the Free Medical Journals website (JMIR is included in this list) [6].

One simple approach to an assessment of the open-access portion of a substantial component of the peer-reviewed research literature can be obtained using PubMed [7], the popular open-access service of the National Library of Medicine in the United States. To test this approach, I carried out (in mid-January of 2003) a small feasibility study, beginning with the PubMed record [8] of Peter Suber's article [3]. PubMed provides a link to *Related Articles*. I sorted the 126 articles that PubMed identified as related to Suber's article by publication date (Suber's article appeared as number 47 on the sorted list). I then tried to access the full text of each article, either directly, via the *click here to read* link provided by PubMed, or via the online electronic journals service of the University of Toronto.

The results for the first 20 articles on the sorted list yielded only 1 article that was openly accessible. In contrast, the University of Toronto Libraries permitted online access (but only for members of the university) to the full text of 10 (50%) of the articles. The remaining articles either were not available in electronic form or had been published in journals to which the University of Toronto Libraries did not have a subscription (such as Lakartidningen, in Swedish). An advantage of this particular list of 20 articles is that all were in different journals, ranging from very well-known ones (such as JAMA) to ones much less well-known, at least to English-speaking readers (such as Lakartidningen). A more-extensive use of this same approach would permit the open-access portion of the research literature covered by PubMed to be estimated, and tracked across time.

No matter which measures of the success of the entire Free Online Scholarship movement are used, it can be anticipated that more and more scholars and researchers will expect to have open access to the articles they wish to read for their own research. And, they will expect to have the option to provide open access to their own research reports.

Moreover, taxpayers are increasingly likely to demand open access to the reports of publicly-funded research. In particular, medical patients and their families, policy-makers, and those with a role in knowledge transfer, can be expected to seek open access to those parts of the primary peer-reviewed research literature that are most clearly relevant to their own needs.

The small feasibility study outlined above also provides a vivid illustration of a major reason why authors of research reports may have less interest in open access than readers of research reports (even though authors and readers are often the same people). Authors associated with universities usually have much more convenient access to the online versions of peer-reviewed research reports than do those who are not associated with universities. So, a strategy to foster open access via author-based self-archiving of peer-reviewed research reports needs to advocate policies that encourage authors to undertake self-archiving. Incentives are likely to be needed. The remainder of this commentary will be focused on one novel model, which will be outlined here as one example of an *incentive model*.

## An Incentive Model

Various ways to use the Internet to facilitate scholarly communication, including communication via research articles, have been outlined by Kling et al [9]. The models preferred by these authors are ones designed to serve as an adjunct to journals and other forms of scholarly communications already in place. The example of an incentive model proposed here is based on this perspective [10]. The model is one where the sponsoring organization is an agency or foundation that provides peer-reviewed grants-in-aid to researchers, but has no interest in launching a new journal. Instead, it would establish an e-print archive, into which only current grantees of the agency would be eligible to post (or submit for posting) reports about the results of research projects or programs that were supported by the agency.

There are several advantages to this model, in comparison with more conventional preprint archives. These include an additional "quality filtering" system that would be involved, in that the research projects or programs would be ones that had recently been peer reviewed by the agency. Only successful applicants would be permitted to post relevant research reports at the e-print archive. These research reports, probably still in the form of preprints, would usually be publicly accessible. The authors/grantees would retain copyright, and, because open access would be based on the copyright holder's consent, there would be no violation of existing copyright law [11]. But, open access to each of the research reports posted at the archive probably should be voluntary, not mandatory. This would permit the opting-out of those authors who may have valid reasons to prefer not to be required to provide open access to any particular research report.

A model in which the funding agency requires that reports about the research it has supported must be openly accessible [12] could be regarded as a *mandatory incentive* model. The somewhat less restrictive *voluntary incentive* model proposed here is designed to foster the concept of open access by "picking the low-hanging fruit," that is, by fostering open access to the research reports of those researchers who are willing to contribute to the agency's e-print archive on a voluntary basis.

In this particular example of a voluntary incentive model, authors/grantees would also be encouraged to submit their posted preprints to an appropriate journal. The most appropriate journals would be ones of satisfactory reputation, which permit authors to retain copyright, and (preferably) provide open access to published articles.

Recipients of research grants from an agency or foundation would probably feel some obligation to post research reports at the agency's e-print archive, and to permit open access to them, if only to provide tangible evidence that their research has been productive. Free online access seems likely to become increasingly popular as evidence continues to accumulate that it can significantly increase the impact of research articles [13].

Also attractive is the prospect that well-designed open-access e-print archives will provide new opportunities for developing methods for evaluating citation rates [13] and for searches of the free, full-text research literature [14]. These methods can be expected to yield novel approaches to assessments of the impact of individual research reports and to explorations of linkages among related research reports.

From the agency's perspective, opportunities to assess, over the longer term, the outcomes of research projects the agency has funded should have considerable appeal. But, to avoid becoming (in effect) a journal publisher, the agency might be well-advised to permit grantees to post research reports at its e-print archive only temporarily. For example, perhaps the lifetime of any particular preprint in the archive should end at either 5 years or when it has been published in an appropriate journal (whichever comes first). Ideally, the agency would also maintain a publicly-accessible database of the research projects and programs that it has funded. It would include, in this database, links to the abstract (and, if possible, to the openly-accessible full text) of any published reports that emanated from these individual projects or programs. And, whether or not the agency decides to become the equivalent of a journal publisher, it should establish e-print archives that are not only openly accessible, but also interoperable [15] (interoperability allows searches across free, full-text research-literature e-print archives; the results can be ranked according to many criteria, eg, by citation impact [14]).

Incentive models of various kinds may become examples of a "little thing that can make a big difference," of the kind described in Malcolm Gladwell's book, "The Tipping Point" [16]. A "tipping point" is "that magic moment when an idea, trend, or social behavior crosses a threshold, tips, and spreads like wildfire." Open access to the peer-reviewed primary biomedical and health research literature has not yet reached a tipping point. Perhaps, via an appropriate mix of strategies of the kind outlined above, it soon will.

## Abbreviations

BOAI: Budapest Open Access Initiative
